# Design and Implementation of Electrochromic Smart Windows with Self-Driven Thermoelectric Power Generation

**DOI:** 10.3390/nano14121027

**Published:** 2024-06-13

**Authors:** Xiaohan Xie, Haining Ji, Lingcan Wang, Shaomei Wang, Qi Chen, Runteng Luo

**Affiliations:** 1 School of Mechanical Engineering and Mechanics, Xiangtan University, Xiangtan 411105, China; 2 School of Physics and Optoelectronics, Xiangtan University, Xiangtan 411105, China

**Keywords:** smart windows, electrochromism, self-driven, thermoelectric power generation

## Abstract

Electrochromic smart windows can achieve controllable modulation of color and transmittance under an external electric field with active light and thermal control capabilities, which helps reduce energy consumption caused by building cooling and heating. However, electrochromic smart windows often rely on external power circuits, which greatly affects the independence and portability of smart windows. Based on this, an electrochromic smart window driven by temperature-difference power generation was designed and implemented. This smart window provides automatic and manual control of the reversible cycle of electrochromic glass from light blue to dark blue according to user requirements and changes in the surrounding environment, achieving adaptive adjustment of visual comfort and reducing energy consumption. The infrared radiation rejection (from 780 to 2500 nm) of the electrochromic smart window is as high as 77.3%, and its transmittance (from 380 to 780 nm) fluctuates between 39.2% and 56.4% with changes in working state. Furthermore, the temperature in the indoor simulation device with electrochromic glass as the window was 15 °C lower than that with ordinary glass as the window after heating with a 250 W Philips infrared lamp for ten minutes. After 2000 cycles of testing, the performance of the smart window was basically maintained at its initial values, and it has broad application prospects in buildings, vehicles, and high-speed rail systems.

## 1. Introduction

With the rapid development of the global economy, energy crises and environmental issues have also emerged. At present, building energy consumption accounts for 40% of total energy consumption and is showing an increasing trend year by year [1,2]. Therefore, promoting building energy conservation and energy-saving renovation of existing buildings has become a key focus for energy conservation, emission reduction, and sustainable development [3,4,5,6]. In addition, windows are the main channel for heat exchange between buildings and the outside world, so it is important to regulate solar radiation through windows to reduce the energy consumption of buildings [7,8,9]. Electrochromic smart windows can not only improve the energy efficiency of buildings, but also adjust the visual comfort of users [10,11,12,13]. Moreover, electrochromism is more controllable and recyclable than photochromism, thermochromism, and gasochromism [6,14,15,16,17], and is more suitable for personalized and market-oriented needs. The weak current principle of the electrochromic smart window determines its controllable performance under the action of external bias. However, adding external biases, such as micro-control for precise control, can also result in additional energy consumption [18,19,20]. Therefore, it is still challenging to reduce energy consumption while improving the control accuracy of self-powered electrochromic smart windows [21,22,23].

The design of electrochromic smart window control systems can be traced back to the early 21st century, when Gugliermetti [24] proposed a systematic control strategy for electrochromic glass to achieve dynamic control of users’ visual comfort. In 2012, an energy storage smart window, consisting of an integrated supercapacitor and electrochromism function in one flexible device, was successfully designed and fabricated [25]. Next, Yeh et al. [26] introduced the concept of self-powered into the design of electrochromic smart windows, providing new ideas for the development of electrochromic glass for flexible devices. Later, a novel integrated gel electrochromic device driven by perovskite solar cells was designed and assembled [27]. This self-powered electrochromic device can quickly change its color state and depth with changes in ambient light intensity, so as to achieve the function of intelligent adjustment of light transmission. And Cannavale et al. [28] summarized the benefits that electrochromic glass can achieve in terms of energy consumption and visual comfort under different control strategies. It can be seen that most electrochromic smart window systems use renewable energy sources, such as solar energy, light energy, or chemical energy, to solve the additional consumption caused by bias circuits. Moreover, control strategies for electrochromic smart windows are constantly updated, and their additional functions are increasing [29,30,31]. Therefore, their social value and related applications are becoming increasingly widespread.

Based on this, an electrochromic smart window system driven by thermoelectric power generation was designed in this study. The use of thermoelectric power generation components solved the energy consumption problems of external bias and electrochromic glass, thereby improving energy utilization efficiency. In addition, under the control of the STM32 controller, the smart window designed in this study utilizes a polling system to monitor the light environment in real time and adjust the working status of the electrochromic glass, so as to achieve self-drive of the electrochromic smart window to adapt to the light environment. Active control can also be achieved by monitoring the status of switch buttons through external interrupts.

## 2. System Summarization

**Figure 1:**
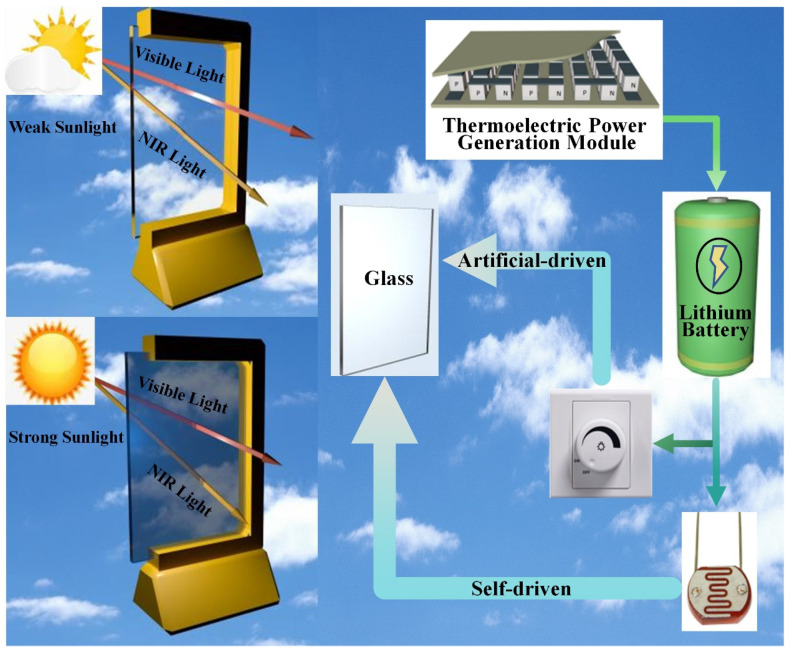
Schematic diagram of the working principle of electrochromic glass driven by thermoelectric power generation.

The self-driven electrochromic smart window utilizes the Seebeck principle to convert thermal energy into potential energy through thermoelectric power generation and stores it in a lithium battery, providing power for the working consumption of the electrochromic glass. The electrochromic glass can achieve a color change from light blue to dark blue through positive pressure (+3.5–+4 V), and a return to the original color through negative pressure (−3.0–−2.5 V). This system can not only manually control the working status of the glass, but also automatically regulate the light transmission performance of the glass according to indoor and outdoor light intensity environments. The schematic diagram of the working principle is shown in Figure 1.

### 2.1. Hardware Scheme

**Figure 2:**
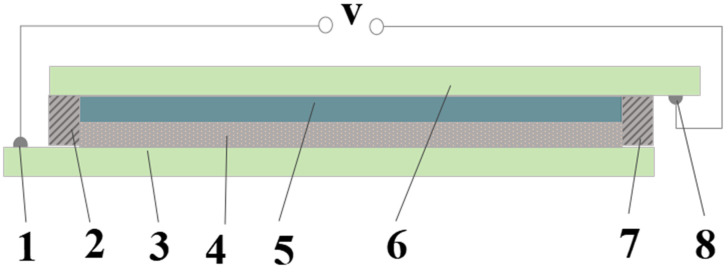
Schematic diagram of electrochromic glass (1 and 8: electrode leads, 2 and 7: encapsulation material, 3 and 6: ITO electrodes, 4: electrolyte layer, and 5: electrochromic layer).

**Figure 3:**
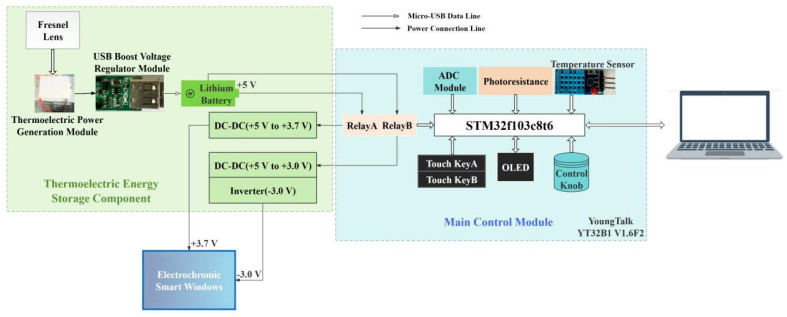
Overall wiring diagram and design principle diagram of the electrochromic smart window driven by thermoelectric power generation.

The self-driven electrochromic smart window is composed of a thermoelectric power generation module, a main control module, and an electrochromic glass module (Zhuhai Kaivo Optoelectronic Technology Co., Ltd., Zhuhai, China). The structural schematic diagram of this electrochromic glass is shown in Figure 2. The electrochromic films we used for the electrochromic glass were nano-films, which determined the performance of the electrochromic glass. The thermoelectric power generation module was assembled using a Fresnel lens, a thermoelectric power generation plate with a heat sink, and a lithium battery. The energy stored by the lithium battery is specifically used for the glass’ working consumption. The forward color changing voltage of the electrochromic glass is 3.5 V to 4 V, and the reverse fading voltage is −3.0 V to −2.5 V. The forward color changing voltage was chosen as 3.7 V, which was relatively easy to obtain. The reverse color changing voltage was converted from 5 V to 3 V through DC–DC modules, and then converted by the inverter. Finally, the required fading working voltage for the electrochromic glass was obtained. Using external DC–DC conversion modules, the required positive and negative voltages for the operation of electrochromic glass can be obtained, and the thermal relay is utilized to realize forward driving and reverse control of the working state of the electrochromic glass. The forward and reverse drive parts are simultaneously connected to the electrochromic glass module, with the negative electrode only grounded, and the positive and negative voltages input through the positive electrode. Negative pressure is mainly achieved by the inverter through positive input. The main control module uses the Yangtao No.1 development board (Model: YoungTalk YT32B1 V1.6F2). The hardware modules used are shown in Figure 3.

### 2.2. Software Scheme

**Figure 4:**
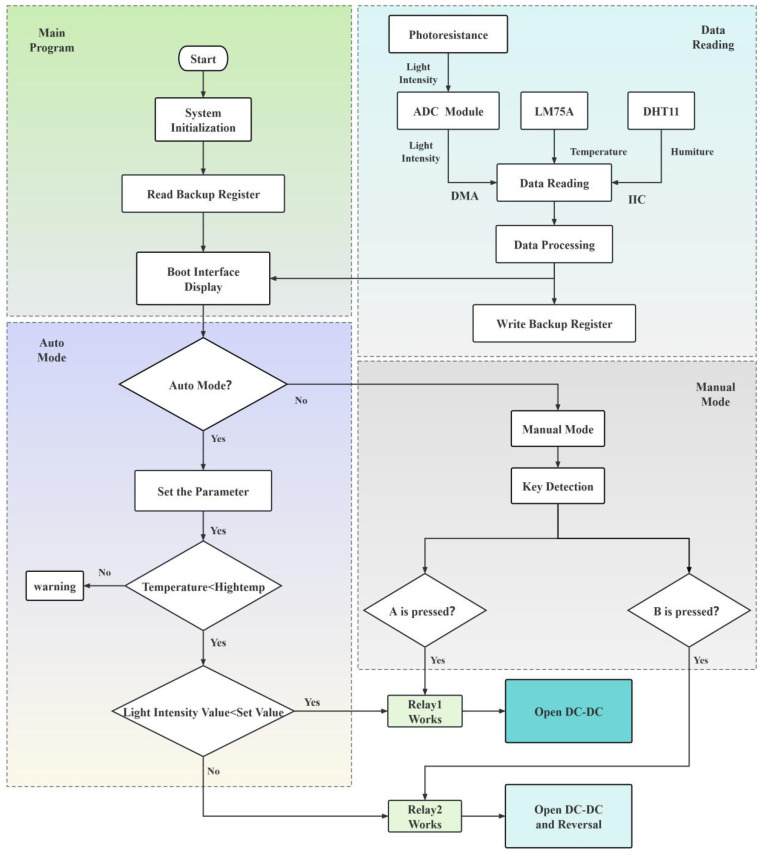
Flow chart of software design.

Auto mode: Self-driven control needs to be sensitive to changes in environmental condition data, so it is a good control strategy to use the polling system to refresh and judge condition data in real time. As shown in Figure 4, after program initialization, external environment data (temperature, humidity, light intensity, etc.) are collected through corresponding sensors, and then data are transmitted through specific communication methods and finally displayed on the OLED after data processing. In addition, the encoder knob can be used to control the switch of the OLED menu interface, and the set value can also be adjusted. External environment data can also be used as conditions for control. For example, the comparison of temperature values is mainly used as an additional function for high temperature alarms. The light intensity value is the control condition for the working state of the electrochromic glass in self-driven mode. When the actual light intensity value is less than the set light intensity value, the thermal relay is turned on with a voltage of +3.7 V to bring the electrochromic glass into a colored state. When the actual light intensity value is greater than the set light intensity value, the thermal relay is turned on to a −3 V voltage to bleach the electrochromic glass.

Manual mode: Active control is a human–computer interaction carried out through the use of buttons as a carrier. In manual mode, after a button interruption occurs, the working status of the thermal relay is controlled to regulate the state of the electrochromic glass. When key A (colored key) is pressed, the thermochromic glass is colored by applying a voltage of +3.7 V, and when key B (bleached key) is pressed, the thermochromic glass is bleached by applying a voltage of −3.0 V.

## 3. Experimental Section

### 3.1. Focusing Experiment Using the Fresnel Lens

The focusing experiment using the Fresnel lens ( Shenzhen Ying Mei Technology Co., Ltd., Shenzhen, China) was conducted on 28 May 2023, in Xiangtan, Hunan Province, China. At that time, the weather was clear and the outdoor temperature was between 26 °C and 37 °C. In order to test the focusing effect of the Fresnel lens and verify its heating effect, an experimental group and a control group were established. The experimental group consisted of two thermoelectric generators (TEP1-142T300, Shenzhen Futian District Xinquan Electronics Technology Co., Ltd., Shenzhen, China) connected in series and combined with heat dissipation fins, as well as a Fresnel lens. The control group consisted of two thermoelectric modules connected in series and bonded with heat dissipation fins. The two sets of experimental components were placed outdoors on a wooden board, and the temperature of the hot end surface of the temperature difference generator was measured at different time points using a TM902C high-temperature rapid electronic thermometer (Jiangsu Kyushu electric heating instrument Technology Co., Ltd., Taizhou, China).

### 3.2. Performance Test of Thermoelectric Power Generation Components

In order to obtain voltage output characteristic curves of the thermoelectric generator under different connection methods, we used the JF966-200 intelligent constant temperature heating platform (Nanjing Detest hardware Franchise Co., Ltd., Nanjing, China) to continuously heat the hot end of the thermoelectric generator under a room temperature environment of 20.2 °C to obtain various temperature differences. Due to the contact between the cold end of the thermoelectric generator and the air, the indoor temperature was considered as the cold end temperature. Using a constant temperature heater to set different hot end temperature values, the expected temperature difference was obtained. The voltage values generated by the thermoelectric generator under different temperature differences were measured using a multimeter, and these data were recorded.

### 3.3. Optical Properties Experiment

The heat-insulating parameters of the electrochromic glass were recorded on a commercial solar-film transmission meter (LS182; Shenzhen Linshang Technology Co., Ltd., Shenzhen, China). Color changes in the electrochromic glass before and after discoloration were measured using a YH1600 haze meter (Shenzhen Sanenshi Technology Co., Ltd., Shenzhen, China) under a D65 standard light source and 10° field of view conditions. The transmittance and corresponding L*, a*, and b* values of the electrochromic glass in two states were obtained. Next, through SQCX software (http://www.3nhcolor.com/product/163-356.html, accessed on 9 July 2023) for data processing, values of x, y, z, R, G, and B of CIE1931 were obtained.

### 3.4. Thermal Performance Test

A thermal insulation device model has only a 12 cm × 12 cm opening in front, and the rest is sealed with aluminum foil. The temperature inside the model is tested using an electronic thermometer. At a room temperature of 16.5 °C, two Philips infrared lamps (250 W) with the same power were used to heat ordinary glass and electrochromic glass insulation devices. The heating distance was 39 cm and the heating time was 10 min. After 10 min, the internal temperatures of the insulation devices placed with ordinary glass and electrochromic glass were recorded.

## 4. Results and Discussion

### 4.1. Thermoelectric Performance

**Figure 5:**
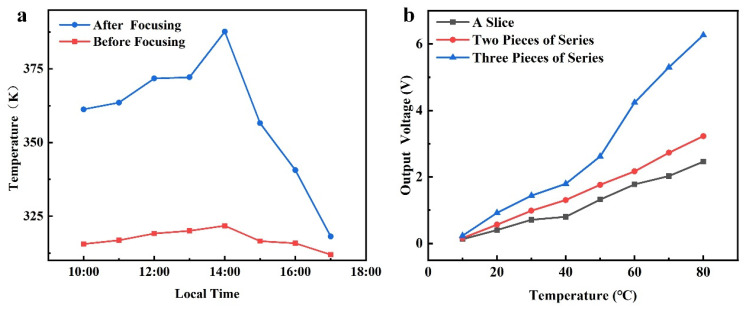
(**a**) Comparison of the temperature of the hot end of the thermoelectric generator before and after concentrating with a Fresnel lens; and (**b**) output characteristic curve of the thermoelectric generator.

According to the Fresnel lens experiment (Figure 5a), the Fresnel lens achieved significant heating effects during focusing, with the most significant heating effect occurring from 1 pm to 3 pm in the afternoon. During this period, the maximum difference in hot end temperatures between the experimental group and the control group reached 65.9 °C.

When the temperature difference generator output a voltage of 0.9–5 V, the voltage for charging and energy storage of the lithium battery could be provided through the boost and stabilization module. According to Figure 5b, when the temperature difference reached 50 °C, the output voltage of a single thermoelectric generator was 1.320 V. Therefore, when a single thermoelectric generator worked, a temperature difference of 50–80 °C achieved the charging and energy storage of lithium batteries. When two thermoelectric generators were connected in series, the output voltage obtained at a temperature difference of 30 °C was 0.986 V. Hence, in the case of two thermoelectric generators connected in series, lithium battery charging and energy storage can be achieved with the temperature difference range of 30–80 °C. When three thermoelectric generators were connected in series, the output voltage at a temperature difference of 20 °C was is 0.922 V, and the output voltage at a temperature difference of 60 °C was 4.239 V. However, when the temperature difference reached 70 °C, the output voltage was 5.303 V, which exceeded the range of the working voltage of the boosting and stabilizing module. So, when three thermoelectric generators are connected in series, the temperature difference between 20 °C and 60 °C can achieve the charging and energy storage of lithium batteries.

According to the output characteristics experiment regarding the thermoelectric generator, when three thermoelectric generators were connected in series, a temperature difference of 20 °C enabled the lithium battery to obtain the required charging voltage. Based on Figure 5a, it can be seen that in clear summer weather, the thermoelectric power generation module easily obtained a temperature difference of 20 °C from 10 a.m. to 4 p.m., and provided the required working voltage to the lithium battery through the boost and stabilization module.

### 4.2. Optical Performance

**Figure 6:**
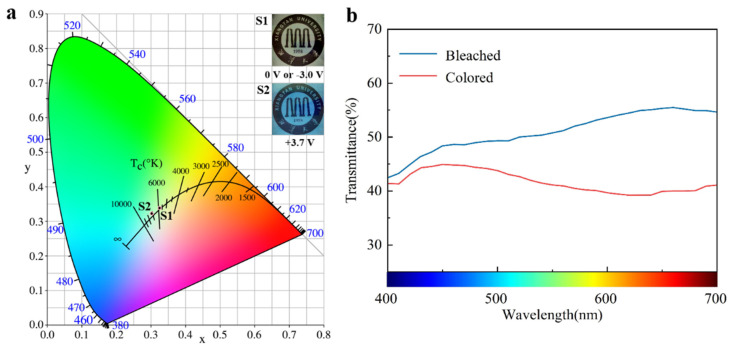
(**a**) CIE chromaticity diagram of electrochromic glass in two states: bleached (S1) and colored (S2); and (**b**) visible transmittance of electrochromic glass in bleached and colored states.

The chromaticity diagram of the electrochromic glass was plotted using CIE1931, and results are shown in Figure 6a. The chromaticity coordinates of the electrochromic glass’ transition from a negative pressure control state or initial state (0.325, 0.338) to a positive pressure control state (0.302, 0.322), and the RGB values of the color gamut horseshoe diagram are recorded in Table 1. From the illustration in Figure 6a, it can be seen that the color of the electrochromic glass gradually changed from light blue to dark blue from the initial state to the working voltage control state of +3.7 V. In addition, the electrochromic glass exhibited good cyclic reversibility during the color gradient process. After 2000 cycles of positive and negative voltage (+3.7 V, −3.0 V), the chromaticity coordinates of electrochromic glass remained basically unchanged. Transmission spectra of the electrochromic glass were almost identical, as shown in Figure 6b. The difference between its UV blocking rate and infrared blocking rate before and after cycling were within 0.5%, as shown in Table 2. A comparison of the electrochromic glass’ and ordinary glass’ optical properties is shown in Table 3. It can be clearly seen that the infrared and ultraviolet blocking rates of the electrochromic glass were significantly higher than those of ordinary glass, and the electrochromic glass has the advantages of dynamically adjusting light and heat compared with ordinary glass. It is more competitive in the market in energy-saving buildings and other fields. These data indicate that the electrochromic glass has good cyclic reversibility and a significant competitive advantage in the smart window market for regulating indoor temperature and visual comfort.

### 4.3. Thermal Performance

**Figure 7:**
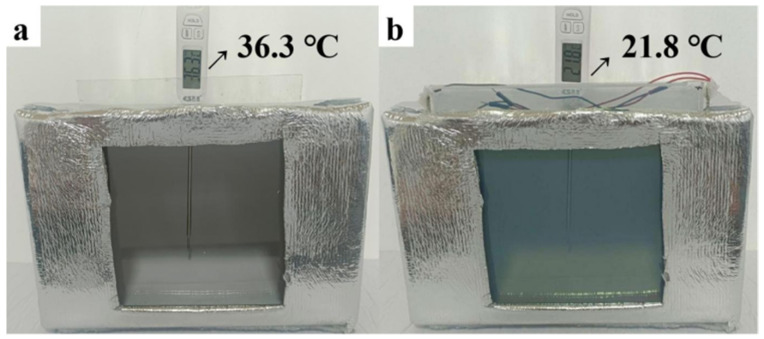
Temperature inside the thermal insulation device model with (**a**) the ordinary glass window and (**b**) the electrochromic glass window after heating for 10 min.

According to Figure 7a,b, it can be seen that after heating with a Philips infrared lamp for ten minutes, the internal temperatures of the insulation devices placed with ordinary glass and the electrochromic glass were 36.3 °C and 21.8 °C, respectively. After repeating the experiment ten times, it was found that under the condition of heating for ten minutes, the temperature inside the electrochromic glass window was about 15 °C lower than that inside the ordinary glass window. These experimental results for thermal performance show that the thermal insulation performance of the electrochromic glass was better than that of ordinary glass.

## 5. Conclusions

In conclusion, electrochromic smart windows with self-driven thermoelectric power generation were designed and implemented. The electrochromic smart window was equipped with temperature difference power generation components, making it self-powered and more energy-efficient. In addition, the designed smart window cannot only achieve self-driving adjustment to adapt to the light environment, but can also be subjectively controlled by humans. It is worth mentioning that after 2000 cycles of alternating positive and negative voltage control, the optical performances of the electrochromic smart window remained stable, indicating its strong cyclic reversibility. Electrochromic smart windows driven by temperature difference power generation have broad application prospects in several fields, such as automotive, high-speed rail, construction, aerospace, flexible devices, etc.

## Figures and Tables

**Table 1:** Coordinate values of bleached and colored states.

State	L*	a*	b*	x	y	z	R	G	B
Bleached	77.49	1.89	4.22	0.32566	0.33870	0.33653	8.46	9.44	9.18
Colored	69.87	−1.61	−3.93	0.30220	0.32294	0.37486	5.55	7.52	8.34

**Table 2:** Infrared and UV blocking rates of electrochromic glass in different cycles of bleached and colored states.

State	Number of Cycles	Infrared Radiation Rejection (780 to 2500 nm)	UV Blocking Rate
Bleached	1	77.3%	91.6%
Bleached	2000	77.3%	91.3%
Colored	1	77.7%	91.0%
Colored	2000	77.7%	90.9%

**Table 3:** Comparison of electrochromic glass and ordinary glass optical properties.

Type of Glass	Infrared Radiation Rejection (780 to 2500 nm)	UV Blocking Rate	Visible Transmittance
Electrochromic glass	77.3%	91.0%	39.2–56.4%
Ordinary glass	16.1%	20.2%	88.2%

## Data Availability

Data are contained within the article.
